# Hypertensive disorders of pregnancy in the United Arab Emirates

**DOI:** 10.3389/frph.2026.1866855

**Published:** 2026-07-30

**Authors:** Ayatullah Hegazy, Minat Allah Alhusami, Ruzaina Sait, Komal Sundeep Hazari, Deemah Harab, Widad Abdelkareem, Muna Tahlak, Fadi Mirza, Lisa Jackson, Yrsa Bergmann Sverrisdottir, William Atiomo, Amar Khamis, Aida J. Azar

**Affiliations:** 1 Graduate Medical Education, Mohammed Bin Rashid University of Medicine and Health Sciences, Dubai Health, Dubai, United Arab Emirates; 2 College of Medicine, Mohammed Bin Rashid University of Medicine and Health Sciences, Dubai Health, Dubai, United Arab Emirates; 3 Obstetrics and Gynecology Department, Latifa Women and Children Hospital, Dubai Health, Dubai, United Arab Emirates; 4 Department of Physiology, School of Health Sciences, University of Iceland, Reykjavík, Iceland; 5 Hamdan Bin Mohammed College of Dental Medicine, Mohammed Bin Rashid University of Medicine and Health Sciences, Dubai Health, Dubai, United Arab Emirates

**Keywords:** epidemiology, gestational hypertension, hypertensive disorders of pregnancy, maternal outcomes, neonatal outcomes, pre-eclampsia, United Arab Emirates

## Abstract

Hypertensive disorders of pregnancy (HDP), including chronic hypertension, gestational hypertension, pre-eclampsia, eclampsia, and HELLP syndrome, are major contributors to adverse maternal and neonatal outcomes. However, epidemiological data from the United Arab Emirates (UAE) remain limited. This study aimed to investigate the prevalence and clinical characteristics of HDP in a large tertiary center, providing evidence from an underrepresented region and a basis for future studies. This six-year retrospective cohort study (2018–2023) was conducted at a tertiary referral center in Dubai, UAE. HDP cases were identified from electronic medical records using ICD-9/10 codes among all deliveries. Maternal and neonatal outcomes were compared by HDP status and World Health Organization (WHO) regions. Multivariate logistic regression was used to identify factors independently associated with HDP. *P* < 0.05 was considered significant. Among 21,415 deliveries, HDP prevalence was 4.7% (*n* = 1,011), while pre-eclampsia prevalence was 2.3%. In multivariable logistic regression, increasing maternal age [OR 1.10 (1.08–1.12)], BMI [OR 1.06 (1.05–1.07)], diabetes [OR 2.31 (1.78–3.00)], renal disease [OR 5.41 (3.12–9.36)], and origin from the Western Pacific Region [OR 1.94 (1.46–2.58)] were independently associated with HDP. Increasing gravidity was associated with lower odds of HDP [OR 0.90 (0.87–0.93)]. Women with HDP delivered earlier, had lower Apgar scores, and had more caesarean delivery. No significant difference in HDP prevalence occurred between UAE nationals and non-UAE nationals. HDP prevalence varied across WHO regions, ranging from 4.3% in the South-East Asian region to 9.1% in the Western Pacific region. In this large multi-ethnic UAE cohort, HDP affected 4.7% of pregnancies and showed regional variation. This study provides one of the first UAE-based estimates of HDP prevalence. Despite prevalence within global estimates, HDP remains a significant public health burden. Strengthened screening and preventive strategies are needed to improve maternal and neonatal outcomes.

## 1 Introduction

Hypertensive disorders of pregnancy (HDP) encompass all forms of high blood pressure that manifest or exacerbate during pregnancy, including chronic hypertension, gestational hypertension (GH), pre-eclampsia with or without severe features, eclampsia, and hemolysis, elevated liver enzymes and low platelet count (HELLP) syndrome. Chronic hypertension is defined as hypertension present before pregnancy or diagnosed before 20 weeks of gestation. Superimposed pre-eclampsia refers to new-onset proteinuria or pre-eclampsia features in a patient with chronic hypertension (1). GH is new-onset hypertension after 20 weeks of gestation without accompanying proteinuria or end-organ damage. When complicated by proteinuria or evidence of end-organ damage, it is referred to as pre-eclampsia, which can progress to eclampsia or to HELLP syndrome. Severity and risk rise along the spectrum from gestational hypertension to eclampsia and HELLP syndrome (1).

Though it is less predictive for gestational hypertension than for pre-eclampsia (2–4), the high and rising prevalence of metabolic syndrome globally, affecting approximately 15%–24% of pregnant women across diverse populations (5), poses a major public health concern. In the United Arab Emirates (UAE), the prevalence of metabolic syndrome among women rises to an estimated 32.7%, with higher rates observed among Emirati women compared to men and other ethnic groups (6).

HDP are estimated to have a mortality rate of 5%-20% among young mothers, often due to complications like HELLP syndrome, disseminated intravascular coagulation, pulmonary oedema, and renal or central nervous system failure (7). Mortality rates among foetuses and infants reach 40%, often due to placental disorders, intrauterine death, and sequelae of prematurity (8). Survivors may have an increased risk of cerebrovascular disease, cardiac disease, diabetes, and dyslipidaemia in mothers, as well as attention-deficit hyperactivity disorder in their offspring (7). Accordingly, in 2012, $2.18 billion were spent on the management of pre-eclampsia in the mother and the neonate within the first 12 months after delivery in the US (9).

A recent publication in Nature Reviews found that HDP showed an unevenly distributed disease burden, with maternal mortality rates being 2.8% in the UK and Ireland, 7.5% in the USA, and 10%-15% in low-and middle-income countries (10). A scoping review published by Hegazy et al. estimated the prevalence of pre-eclampsia in the Middle East to range from 0.17 to 5.0%, but no studies were identified in the UAE (11). Despite the UAE's higher prevalence of key risk factors for HDP, there remains a lack of published data on the prevalence of HDP in the country (12). Furthermore, evidence from the UAE remains scarce despite the country's unique multinational population and healthcare landscape. The co-existence of UAE nationals and non-UAE nationals from diverse geographic backgrounds within a single healthcare environment provides a unique opportunity to explore variation in HDP burden and clinical characteristics while minimizing the heterogeneity inherent in cross-country comparisons. This study aimed to investigate the prevalence and clinical characteristics of women with HDP in the main governmental obstetrics and gynecology hospital in Dubai, the primary tertiary referral center for high-risk pregnancies and complex obstetric cases, providing a valuable opportunity to generate epidemiologic data from a large and diverse obstetric population. By examining the epidemiology of HDP in the local population, the study seeks to raise awareness, inform screening strategies, and support the targeted interventions to improve health outcomes of both mothers with HDP and their offspring.

## 2 Methods

### 2.1 Study design and setting

This six-year retrospective cohort study was conducted in accordance with the STROBE (Strengthening the Reporting of Observational Studies in Epidemiology) guidelines at Latifa Women and Children Hospital (LWCH) (13). LWCH is the main obstetrics and gynaecology governmental hospital in Dubai, UAE, and serves as the primary tertiary referral centre for severe and complicated obstetric cases from all Dubai hospitals. At the same time, it provides routine obstetric care to a large, ethnically diverse population comprising both UAE nationals and non-UAE nationals. This population therefore represents a broad spectrum of pregnancies ranging from routine obstetric care to complex high-risk cases within Dubai and the UAE.

### 2.2 Data extraction

Data was extracted from Salama, the electronic medical records system (EMR), to estimate the prevalence of HDP in women who delivered at LWCH from 1 January 2018 to 31 December 2023. All women who delivered during this period and their offspring were included. Women who did not deliver at LWCH or had missing records were excluded. The EMR birth registry identified eligible cases. Women with HDP were identified using ICD-9 and ICD-10 codes extracted from the electronic medical record system. Formal validation of ICD-based case identification through detailed chart review was beyond the scope of this study. The clinical definitions of chronic hypertension, gestational hypertension, and pre-eclampsia used at LWCH are consistent with the American College of Obstetricians and Gynaecologists (ACOG) Practice Bulletin and Guidelines (14). HDP comprised a spectrum of hypertensive conditions occurring during pregnancy, including chronic hypertension, gestational hypertension, pre-eclampsia, eclampsia, and HELLP syndrome. Hypertension was defined as systolic blood pressure ≥140 mmHg and/or diastolic blood pressure ≥90 mmHg. Gestational hypertension referred to new-onset hypertension developing after 20 weeks of gestation without proteinuria or severe features. Pre-eclampsia was defined as hypertension arising after 20 weeks of gestation in the presence of proteinuria and/or maternal organ dysfunction. Chronic hypertension referred to hypertension diagnosed before pregnancy or before 20 weeks of gestation.

As specified by physicians at LWCH the following codes were applied to capture all women with HDP: Hypertension complication pregnancy, childbirth and puerperium (642), Essential hypertension (401), Gestational hypertension without significant proteinuria (O13), Pre-eclampsia (O14), Unspecified maternal hypertension (O16), Pre-existing hypertension complicating pregnancy, childbirth and the puerperium (O10), Pre-existing hypertension with pre-eclampsia (O11), Essential hypertension (I10) and Secondary hypertension (I15).

Demographic, clinical, and delivery data were obtained from the EMR. These included the maternal age, nationality, past medical history, maternal weight in kilograms (at diagnosis), maternal BMI, number of deliveries, systolic and diastolic blood pressure at diagnosis, development of eclampsia and foetal growth restriction, Apgar scores at 1 and 5 min, gestational age at delivery, baby's birth weight in kilograms, and delivery mode.

### 2.3 Ethical approval

Ethical approval was obtained from the Mohammed Bin Rashid University IRB (MBRU IRB-2023-62). IRB approval was also granted from Dubai Scientific Research Ethics committee (DSREC) (reference number DSREC-12/2023_04). As there was no direct patient contact, patient consent was waived as per the IRB.

### 2.4 Data analysis and statistics

All patient records, including offspring and delivery files, were de-identified and assigned unique study codes to ensure confidentiality. Duplicate offspring records were removed. Duplicate delivery records, which indicated multiple births within the same pregnancy, were collapsed so that each delivery contributed only once to maternal analyses. Mothers with multiple pregnancies during the study period were included, and each delivery was treated as a separate observation. No records with missing information were noted. HDP status was assigned at the delivery level based on ICD-9/10 codes recorded for that delivery. The prevalence of HDP and pre-eclampsia was calculated at the delivery level as the number of deliveries complicated by HDP or pre-eclampsia divided by the total number of deliveries included in the study.

Offspring data were analysed using all available records; however, babies with missing Apgar scores, birth weight, or gestational age were excluded from the relevant analysis. Patients were further sub-grouped by nationality (UAE nationals vs. non-UAE nationals). Given Dubai's diverse population, patients were also compared by region of origin, categorized according to World Health Organization (WHO) regions (Supplementary Table 1).

Categorical variables were summarized as frequencies and percentages, and differences between groups were assessed using the Chi-square test or Fisher's exact test when expected cell counts were small. Continuous variables were summarized as means with standard deviations if normally distributed, or as medians with interquartile ranges when not normally distributed. Normality was assessed using the Kolmogorov–Smirnov test together with graphical methods (histograms and Q–Q plots). Depending on the distribution, comparisons between independent groups were conducted using either the independent-samples T-test or the Mann–Whitney U test. Multivariate logistic regression was performed with HDP status as the dependent variable to identify factors independently associated with HDP. Adjusted odds ratio (OR) and 95% confidence intervals (CI) were reported. Statistical significance was defined as *p* < 0.05. All analyses were performed using the Statistical Package for Social Sciences (SPSS, version 29.0).

## 3 Results

**Figure 1:**
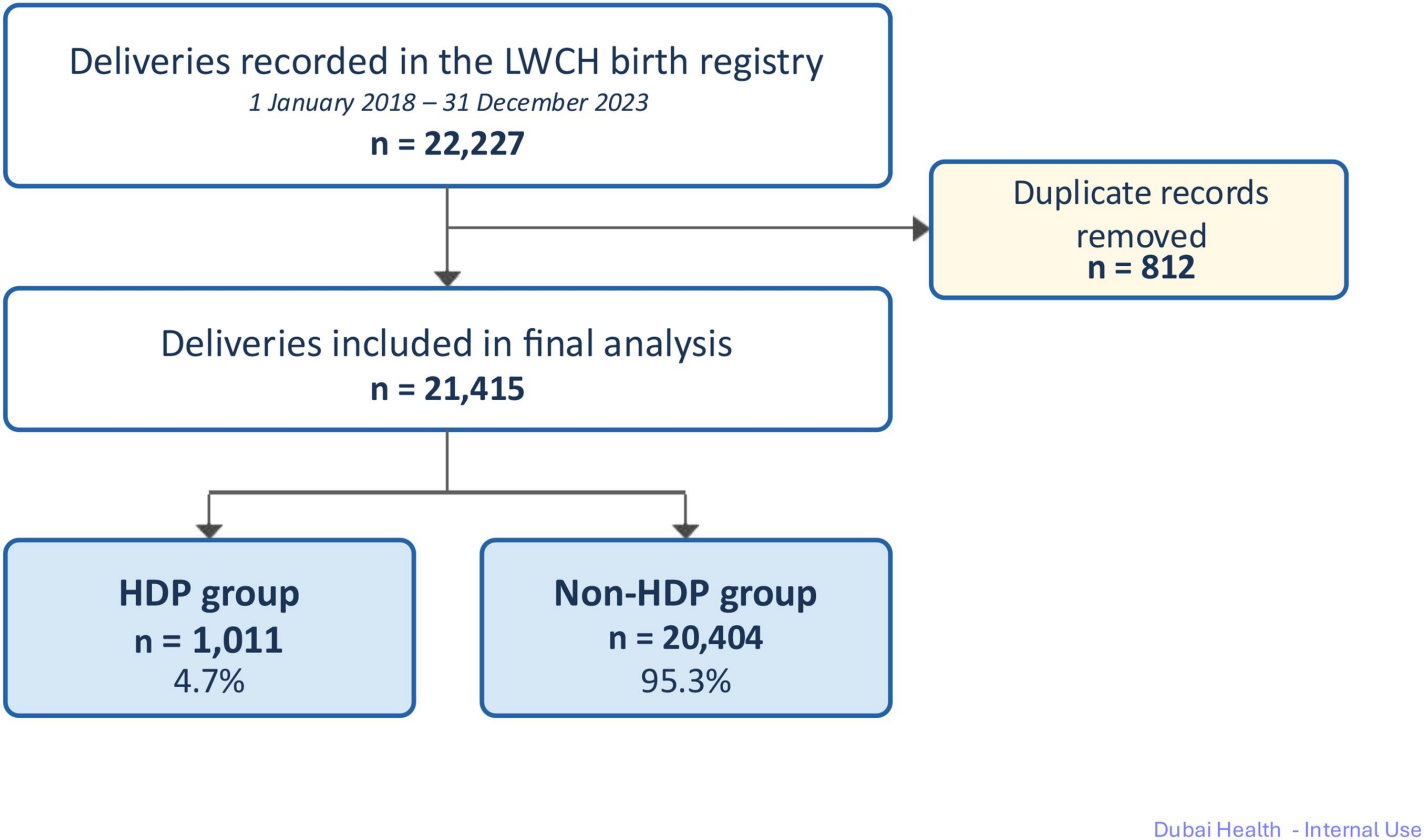
Participant selection flow diagram. Legend- Deliveries recorded at Latifa Women and Children Hospital (LWCH), Dubai, UAE, between 1 January 2018 and 31 December 2023 were screened for eligibility (*N* = 22,227). After removal of duplicate records (*n* = 812), 21,415 deliveries were included in the final analysis and classified as either hypertensive disorders of pregnancy (HDP; *n* = 1,011) or non-HDP (*n* = 20,404).

A total of 22,227 pregnancies recorded at Latifa Women and Children Hospital (LWCH) over a 6-year period, between 1 January 2018 and 31 December 2023 were screened for eligibility (Figure 1). Following removal of 812 duplicate records, 21,415 unique pregnancies were included in the final analysis. Among these, 1,011 pregnancies (4.7%) were complicated by hypertensive disorders of pregnancy (HDP) (Figure 1). Of the total pregnancies, 20,666 (96.5%) were singleton pregnancies, 697 (3.3%) were twin pregnancies, 47 (0.2%) were triplet pregnancies, and 5 (<0.1%) involved quadruplets or higher-order multiples.

### 3.1 Prevalence of HDP and Pre-eclampsia

**Figure 2:**
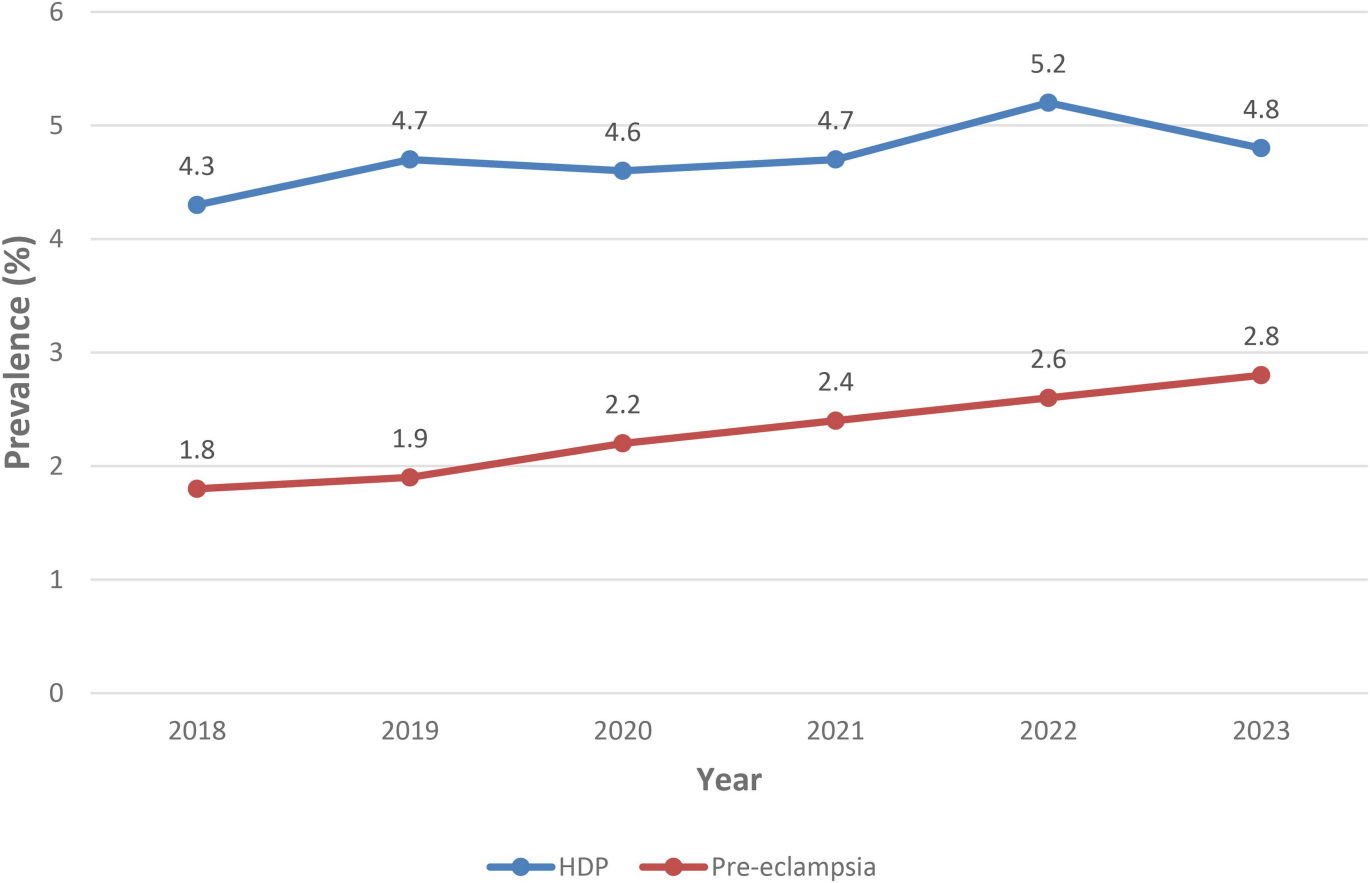
Annual prevalence of hypertensive disorders of pregnancy and pre-eclampsia (*N* = 21,415). Legend- Trends in hypertensive disorders of pregnancy (HDP) and pre-eclampsia from 2018 to 2023. Over the 6-year study period, the prevalence of HDP (orange line) remained relatively stable between 4 and 5% (overall prevalence: 4.7%). In contrast, the prevalence of pre-eclampsia (blue line) showed a gradual increase rising from 1.8% in 2018 to 2.8% in 2023 (overall prevalence: 2.3%).

 Figure 2 shows the annual prevalence of HDP, which remained relatively stable over the 6-year study period (2018-2023), with an overall study-period prevalence of 4.7%. The prevalence ranged from 4.3% in 2018 and peaking at 5.2% in 2022, with no significant change in the prevalence during the COVID-19 pandemic. On the other hand, the prevalence of pre-eclampsia increased gradually, starting at 1.8% in 2018 and reaching 2.8% in 2023, with an overall prevalence of 2.3% (Figure 2).

### 3.2 Characteristics of women with HDP

 Table 1 shows that women with HDP were significantly older than those without HDP [mean age 34.6 (SD 5.8) vs. 31.6 (SD 5.8) years, *p* < 0.001] and had a higher mean gravidity [3.8 (2.7) vs. 3.6 (2.4), *p* = 0.014], while mean parity was comparable between the two groups (2.0 vs. 1.9). Women with HDP had significantly higher mean BMI (kg/m^2^) [33.8 (6.3) vs. 31.0 (5.9), *p* < 0.001] and blood pressure readings (*p* < 0.001), as well as significantly higher rates of comorbidities, including PCOS [16 (1.6%) vs. 188 (0.9%), *p* = 0.033], hypertension [419 (41.4%) vs. 107 (0.5%), *p* < 0.001], diabetes mellitus [101 (10.0%) vs. 535 (2.6%), *p* < 0.001], renal disease [27 (2.7%) vs. 61 (0.3%), *p* < 0.001], and hyperthyroidism [19 (1.9%) vs. 243 (1.2%), *p* = 0.043] (Table 1).

**Table 1:** Demographic and clinical characteristics of women with and without hypertensive disorders of pregnancy in a large obstetric cohort from dubai, United Arab Emirates (*N* = 21,415).

Variable	HDP Present (*n* = 1011)	HDP Absent (*n* = 20404)	*P*-value
Maternal age, mean (SD) (years)	34.6 (5.8)	31.6 (5.8)	<0.001
Gravida, mean (SD)	3.8 (2.7)	3.6 (2.4)	0.014
Parity, mean (SD)	2.0 (2.1)	1.9 (1.9)	0.128
Maternal BMI, mean (SD) (kg/m^2^)	33.8 (6.3)	31.0 (5.9)	<0.001
Systolic blood pressure, mean (SD) (mm/Hg)	131.9 (14.1)	113.6 (10.5)	<0.001
Diastolic blood pressure, mean (SD) (mm/Hg)	78.0 (10.9)	68.1 (7.8)	<0.001
Gestational age, mean (SD) (weeks)	35.6 (3.3)	37.5 (2.9)	<0.001
Baby weight, mean (SD) (kg)	2.5 (0.8)	2.9 (0.6)	<0.001
Delivery mode			
Caesarean delivery, *n* (%)	636 (62.9)	7482 (36.7)	<0.001
Vaginal, *n* (%)	375 (37.1)	12922 (63.3)	
Apgar score at 1 min, mean (SD)	7.5 (1.8)	8.1 (1.4)	<0.001
Apgar score at 5 min, mean (SD)	8.8 (1.6)	9.2 (1.2)	<0.001
Maternal Comorbidities:			
PCOS, *n* (%)	16 (1.6)	188 (0.9)	0.033
Hypertension, *n* (%)	419 (41.4)	107 (0.5)	<0.001
Diabetes, *n* (%)	101 (10.0)	535 (2.6)	<0.001
Renal disease, *n* (%)	27 (2.7)	61 (0.3)	<0.001
Antiphospholipid syndrome, *n* (%)	18 (1.8)	256 (1.3)	0.099
Hyperthyroidism, *n* (%)	19 (1.9)	243 (1.2)	0.043
Foetal growth restriction, *n* (%)	6 (0.6)	16 (0.1)	<0.001

HDP, hypertensive disorders of pregnancy; BMI, body mass index (kg/m2); PCOS, polycystic ovarian syndrome; n, number; SD, standard deviation; kg, kilogram.

Foetal growth restriction was significantly more frequent among hypertensive pregnancies (0.6% vs. 0.1%, *p* < 0.001). Neonates delivered to women with HDP also had lower mean gestational ages [35.6 (SD 3.3) weeks vs. 37.5 (SD 2.9%) weeks, *p* < 0.001] and higher rates of caesarean delivery [636 (62.9%) vs. 7482 (36.7%), *p* < 0.001]. Apgar scores were found to be poorer in HDP cases, with lower mean scores at 1 min [7.5 (SD 1.8) vs. 8.1 (SD 1.4), *p* < 0.001] and 5 min [8.8 (1.6) vs. 9.2 (1.2), *p* < 0.001] (Table 1).

### 3.3 UAE nationals vs. Non-UAE nationals

No significant difference in HDP prevalence was observed between UAE nationals and non-UAE nationals (4.6% vs. 4.6%, *p* = 0.106). Analysis of pregnancies stratified by nationality revealed subtle demographic and clinical differences between the groups. Table 2 shows that among pregnancies complicated by HDP, UAE nationals were older [mean age 35.1 (SD 6.1) vs. 34.0 (SD 5.4) years, *p* = 0.002] and had higher mean BMI kg/m^2^ [34.4 (SD 6.4) vs. 32.9 (SD 6.0), *p* < 0.001], gravidity [4.5 (SD 2.9) vs. 2.9 (1.9), *p* < 0.001], parity [2.6 (2.3) vs. 1.4 (1.5)], *p* < 0.001), and chronic hypertension rates [268 (47.5%) vs. 151 (33.8%), *p* < 0.001] compared to non-UAE nationals (Table 2). Diabetes mellitus, renal disease, antiphospholipid syndrome, and hyperthyroidism were also slightly more common in UAE nationals with HDP (64 (11.3%), 19 (3.4%),11 (2.0%), and 12 (2.1%) than in non-UAE nationals (37 (8.3%), 8 (1.8%), 7 (1.6%), and 2 (1.6%)), but these differences did not achieve statistical significance (Table 2). Offspring of non-UAE nationals had significantly lower mean Apgar scores at 1 min [7.6 (SD 1.7) vs. 7.4 (SD 1.8), *p* = 0.041] and at 5 min [8.9 (SD 1.4) vs. 8.7 SD 1.7], *p* = 0.035) and younger mean gestational age [35.9 (SD 3.2) vs. 35.3 (SD 3.5) weeks, *p* = 0.018], while baby weight was similar [2.5 (SD 0.8) vs. 2.4 (SD 0.9) kg] between the groups (Table 2).

**Table 2:** Comparison of clinical characteristics of UAE nationals and non-UAE nationals with hypertensive disorders of pregnancy (*n* = 1011).

Variable	HDP Present		
	UAE Nationals	Non-UAE nationals	*P*-value
	(*n* = 564)	(*n* = 447)	
	Mean (SD)	Mean (SD)	
Maternal age (years)	35.1 (6.1)	34.0 (5.4)	0.002
Gravida	4.5 (2.9)	2.9 (1.9)	<0.001
Parity	2.6 (2.3)	1.4 (1.5)	<0.001
Maternal BMI (kg/m^2^)	34.4 (6.4)	32.9 (6.0)	<0.001
Gestational age (weeks)	35.9 (3.2)	35.3 (3.5)	0.018
Apgar score at 1 min	7.6 (1.7)	7.4 (1.8)	0.041
Apgar score at 5 min	8.9 (1.4)	8.7 (1.7)	0.035
Baby weight (kg)	2.5 (0.8)	2.4 (0.9)	0.112

	**n (%)**	**n (%)**	***P*-value**
Delivery mode			<0.001
Caesarean delivery	343 (60.8)	216 (48.3)	
Vaginal	221 (39.2)	231 (51.7)	
Maternal Comorbidities:			
PCOS	8 (1.4)	8 (1.4)	0.801
Hypertension	268 (47.5)	151 (33.8)	<0.001
Diabetes	64 (11.3)	37 (8.3)	0.114
Renal disease	19 (3.4)	8 (1.8)	0.168
Antiphospholipid syndrome	11 (2.0)	7 (1.6)	0.812
Hyperthyroidism	12 (2.1)	7 (1.6)	0.643

UAE, the United Arab Emirates; n, number; SD, standard deviation; BMI, body mass index (kg/m^2^); PCOS, polycystic ovarian syndrome.

*P*-value (<0.05) is considered statistically significant.

### 3.4 Multivariate logistic regression analysis

Multivariable logistic regression analysis identified several factors independently associated with HDP after adjustment for potential confounders (Table 3). Each one-year increase in maternal age was associated with a 10% increase in the odds of HDP [OR 1.10 (1.08–1.12)], while each one-unit increase in BMI was associated with a 6% increase in the odds of HDP [OR 1.06 (1.05–1.07)]. Diabetes [OR 2.31 (1.78–3.00)] and renal disease [OR 5.41 (3.12–9.36)] were associated with 2.3-fold and 5.4-fold higher odds of HDP, respectively (Table 3). Women originating from countries within the Western Pacific Region had 94% higher odds of HDP compared with women from all other WHO regions combined [OR 1.94 (1.46–2.58)]. In contrast, each additional pregnancy was associated with a 10% reduction in the odds of HDP [OR 0.90 (0.87–0.93)]. PCOS and antiphospholipid syndrome were not independently associated with HDP (Table 3). The overall model was statistically significant (Omnibus *χ*^2^ = 458.7, df = 8, *p* < 0.001). The Nagelkerke R^2^ was 0.074, indicating that the variables included in the model explained approximately 7.4% of the variation in HDP status (Table 3).

**Table 3:** Multivariate logistic regression analysis of factors independently associated with hypertensive disorders of pregnancy (*N* = 21,415).

Variable	Coefficient (*β*)	Adjusted Odds Ratio	95% CI	*p*-value
Maternal age (per year increase)	0.095	1.10	1.08–1.12	<0.001
Maternal BMI (per kg/m^2^ increase)	0.057	1.06	1.05–1.07	<0.001
Gravida (per pregnancy increase)	−0.109	0.90	0.87–0.93	<0.001
Western Pacific Region (vs. all other WHO regions)	0.663	1.94	1.46–2.58	<0.001
PCOS	0.326	1.39	0.79–2.44	0.261
Diabetes	0.836	2.31	1.78–3.00	<0.001
Renal disease	1.687	5.41	3.12–9.36	<0.001
Antiphospholipid syndrome	0.351	1.42	0.85–2.38	0.181

CI, confidence interval; BMI, body mass index (kg/m2); WHO, World Health Organization, PCOS, polycystic ovarian syndrome. Maternal age, BMI, and gravidity were entered as continuous variables. Model fit statistics: Omnibus χ^2^ = 458.7, df = 8, p < 0.001; Nagelkerke R^2^ = 0.074; −2 Log Likelihood = 6820.4.

### 3.5 Prevalence by WHO region

HDP prevalence varied across the WHO regions of origin (Table 4). The highest prevalence was seen among women from the WPR (*n* = 70, 9.1%), followed by the AMR (*n* = 6, 9.0%) and AFR (*n* = 97, 7.2%). Prevalence was lower among women from EUR (*n* = 19, 4.7%), EMR (*n* = 748, 4.4%) and in SEAR (*n* = 68, 4.3%) (Table 4).

**Table 4:** Maternal characteristics of women with and without hypertensive disorders of pregnancy by WHO region of origin (*N* = 21,415).

Maternal characteristics	EMR		EUR		AFR		SEAR		AMR		WPR	
	HDP Present	HDP Absent	HDP Present	HDP Absent	HDP Present	HDP Absent	HDP Present	HDP Absent	HDP Present	HDP Absent	HDP Present	HDP Absent
	(*n* = 748)	(*n* = 16437)	(*n* = 19)	(*n* = 387)	(*n* = 97)	(*n* = 1254)	(*n* = 68)	(*n* = 1515)	(*n* = 6)	(*n* = 61)	(*n* = 70)	(*n* = 700)
Prevalence (%)^a^	4.4		4.7		7.2		4.3		9.0		9.1	
Maternal age (years)	34.6 (6.1)	31.5 (5.9)	31.9 (5.3)	33.0 (5.3)	33.6 (5.6)	31.2 (5.5)	35.0 (4.3)	31.9 (5.5)	29.2 (4.9)	31.1 (5.5)	37.0 (4.2)	34.1 (4.8)
Gravida	4.1 (2.8)	3.8 (2.4)	2.1 (1.8)	2.7 (1.8)	3.1 (2.3)	3.0 (2.1)	2.9 (1.7)	2.5 (1.5)	2.3 (1.6)	2.8 (1.8)	2.5 (1.4)	2.4 (1.4)
Parity	2.3 (2.2)	2.1 (1.9)	0.8 (1.3)	1.2 (1.4)	1.5 (1.8)	1.5 (1.7)	1.3 (1.3)	1.1 (1.1)	0.8 (1.3)	1.3 (1.5)	1.2 (1.2)	1.2 (1.3)
Maternal BMI (kg/m^2^)	34.1 (6.3)	31.3 (6.0)	31.8 (6.5)	30.0 (5.0)	34.2 (7.0)	30.8 (6.1)	32.9 (4.7)	29.8 (4.7)	34.0 (5.4)	30.7 (5.0)	31.0 (5.1)	28.6 (4.4)
Gestational age (weeks)	35.9 (3.2)	37.7 (2.7)	35.3 (3.6)	37.6 (3.3)	35.5 (3.5)	37.5 (3.4)	35.2 (3.3)	37.3 (3.3)	34.0 (3.3)	37.2 (3.4)	34.1 (3.9)	36.2 (4.3)

Values are presented as mean (SD).

a Prevalence (%) represents the proportion of women with HDP among all deliveries within each WHO region. HDP, hypertensive disorders of pregnancy; SD, standard deviation; BMI, body mass index; WHO, World Health Organization; EMR, Eastern Mediterranean Region; EUR, European Region; AFR, African Region; SEAR, South-East Asian Region; AMR, Region of the Americas; WPR, Western Pacific Region.

#### 3.5.1 Eastern Mediterranean region (EMR)

Women with HDP from the EMR had a mean maternal age of 34.6 years (SD 6.1) and the highest gravidity (4.1, SD 2.8) and parity (2.3, SD 2.2) among all the WHO regions (Table 4). They also exhibited the highest burden of hypertension 327 (43.7%), diabetes 82 (11.0%), and renal disease 24 (3.2%), while PCOS was relatively common 10 (1.3%) (Table 5). The prevalence of HDP in the EMR was relatively low (4.4%), despite the relatively high prevalence of comorbidities observed in the subgroup (Table 4). Neonatal outcomes were comparatively better than in other regions, with the highest mean gestational age [35.9 (SD 3.2) weeks] across WHO regions (Table 4), despite having the highest rate of foetal growth restriction (0.8%). Caesarean delivery rates were high 455 (61.4%), but lower than those observed in the AFR or WPR (Table 5).

**Table 5:** Obstetric outcomes and maternal comorbidities among women with and without hypertensive disorders of pregnancy by WHO region of origin (*N* = 21,415).

Obstetric outcomes and comorbidities	EMR		EUR		AFR		SEAR		AMR		WPR	
	HDP Present	HDP Absent	HDP Present	HDP Absent	HDP Present	HDP Absent	HDP Present	HDP Absent	HDP Present	HDP Absent	HDP Present	HDP Absent
	(*n* = 748)	(*n* = 16437)	(*n* = 19)	(*n* = 387)	(*n* = 97)	(*n* = 1254)	(*n* = 68)	(*n* = 1515)	(*n* = 6)	(*n* = 61)	(*n* = 70)	(*n* = 700)
Delivery mode												
C-section	455 (61.4)	5910 (36.6)	12 (63.2)	158 (41.1)	71 (74.0)	524 (42.4)	44 (64.7)	574 (38.2)	4 (66.7)	28 (46.7)	47 (67.1)	270 (39.0)
Vaginal	286 (38.6)	10228 (63.4)	7 (36.8)	226 (58.9)	25 (26.0)	712 (57.6)	24 (35.3)	927 (61.8)	2 (33.3)	32 (53.3)	23 (32.9)	423 (61.0)
Maternal comorbidities												
PCOS	10 (1.3)	148 (0.9)	0 (0)	0 (0)	3 (3.1)	8 (0.6)	3 (4.4)	24 (1.6)	0 (0)	0 (0)	0 (0)	1 (0.1)
Hypertension	327 (43.7)	80 (0.5)	4 (21.1)	0 (0)	34 (35.1)	6 (0.5)	25 (36.8)	11 (0.7)	0 (0)	1 (1.6)	29 (41.4)	9 (1.3)
Diabetes	82 (11.0)	459 (2.8)	0 (0)	4 (1.0)	9 (9.3)	24 (1.9)	5 (7.4)	44 (2.9)	0 (0)	2 (3.3)	5 (7.1)	2 (0.3)
Renal disease	24 (3.2)	54 (0.3)	0 (0)	0 (0)	0 (0)	4 (0.3)	2 (2.9)	0 (0)	0 (0)	0 (0)	1 (1.4)	3 (0.4)
APS	14 (1.9)	227 (1.4)	1 (5.3)	3 (0.8)	2 (2.1)	10 (0.8)	0 (0)	12 (0.8)	0 (0)	0 (0)	1 (1.4)	4 (0.6)
Hyperthyroidism	15 (2.0)	202 (1.2)	0 (0)	4 (1.0)	2 (2.1)	19 (1.5)	1 (1.5)	13 (0.9)	1 (1.6)	2 (3.3)	0 (0)	3 (0.4)

Values are presented as *n* (%). Percentages were calculated within each WHO region and HDP-group. n, number; HDP, hypertensive disorders of pregnancy; PCOS, polycystic ovary syndrome; APS, antiphospholipid syndrome; WHO, world health organization; EMR, Eastern Mediterranean region; EUR, European region; AFR, African region; SEAR, South-East Asian region; AMR, region of the Americas; WPR, western pacific region.

#### 3.5.2 European region (EUR)

Women with HDP in EUR were among the youngest across the WHO regions (31.9 years, SD 5.3), and prevalence of HDP in this region was relatively low (4.7%) (Table 4). Rates of maternal PCOS, chronic hypertension, diabetes and renal disease were among the lowest in this group (0 (0%), 4 (21.1%), 0 (0%) and 0 (0%), respectively) (Table 5). Only 1 patient (5.3%) had antiphospholipid syndrome (Table 5). Neonatal outcomes were poor, with a mean gestational age of 35.3 weeks (SD 3.6) (Table 4). Caesarean-section deliveries were comparably higher than other regions (12, 63.2%) (Table 5).

#### 3.5.3 African region (AFR)

The prevalence of HDP was relatively high among women from the AFR, with 97 cases (7.2%) (Table 4). Women with HDP from this region had a mean maternal age of 33.6 years (SD 5.6) and the highest mean maternal BMI 34.2 kg/m^2^, (SD 7.0) among all WHO regions (Table 4). They displayed among the highest rates of comorbid PCOS 3 (3.1%), hypertension 34 (35.1%), and diabetes 9 (9.3%) (Table 5). However, renal disease and antiphospholipid syndrome were less common (0% and 2.1%, respectively) (Table 5). Neonatal outcomes showed a mean gestational age at delivery of 35.5 weeks (SD 3.5), and the highest caesarean delivery rate among all WHO regions 71 (74.0%) (Table 4).

#### 3.5.4 South-East Asia region (SEAR)

SEAR demonstrated the lowest HDP prevalence (4.3%). However, affected women with HDP were among the oldest, with a mean maternal age of 35.0 years (SD 4.3) (Table 4), and had the highest rates of PCOS (4.4%) (Table 5). Among women with HDP, the mean maternal BMI was 32.9 (SD 4.7), while hypertension and diabetes were present in 36.8% and 7.4%, respectively (Table 5). Neonatal outcomes were less favorable, with a low mean gestational age at delivery (35.2 weeks), and higher caesarean delivery rate (66.7%) (Table 4).

#### 3.5.5 Region of the Americas (AMR)

HDP prevalence was relatively high in AMR (9.0%), however, the number of cases was small (*n* = 6) and prevalence estimates should therefore be interpreted with caution (Table 4). Women with HDP in this group had the lowest mean (29.2 years, SD 4.9) years (SD 4.9) and among the highest mean maternal BMI values (34.0 kg/m^2^, SD 5.4) (Table 4). Despite the small sample, neonatal outcomes appeared less favourable, with the lowest mean gestational age at delivery (34 weeks, SD 3.3) (Table 4), and one of the highest caesarean deliveries rates (47, 66.7%) (Table 5).

#### 3.5.6 Western Pacific region (WPR)

WPR showed the highest HDP prevalence among all WHO regions, with 70 cases (9.1%). Women with HDP in this subgroup had the highest mean maternal age (37.0 years, SD 4.2) (Table 4). They had high caesarean delivery rates (47 cases, 67.1%), and mean gestational age at delivery was relatively low (34.1 weeks, SD 3.9). Comorbid hypertension (*n* = 29 cases, 41.4%) and diabetes (*n* = 5, 7.1%) were also common in women with HDP (Table 5). The mean maternal BMI kg/m^2^ was 31.0 (SD 5.1), and rates of PCOS, renal disease, and antiphospholipid syndrome were low (0%, 1.4%, and 1.4%, respectively) (Table 5).

## 4 Discussion

This retrospective cohort study is among the first to report a UAE-based estimate of HDP prevalence using data from the birth registry of Latifa Women and Children Hospital (LWCH), a major tertiary referral centre in Dubai. As the main governmental obstetrics and gynaecology hospital in Dubai, LWCH serves a large and ethnically diverse obstetric population. Its central location and accessibility, including to women without private health insurance, likely contribute to its broad and diverse obstetric population. This diversity provided a valuable setting to examine HDP prevalence and maternal and neonatal characteristics across WHO regions of origin within a single healthcare setting. Although HDP prevalence has been reported in individual countries, comparative data across WHO regions of origin within a single healthcare setting remain limited. However, the prevalence estimate reported in this study should be interpreted as a hospital-based estimate rather than a nationally representative estimate for the UAE.

Several maternal characteristics and adverse pregnancy outcomes were more frequently observed among women with HDP in our study population. Women with HDP were significantly older, delivered at earlier gestational ages, and were more likely to undergo caesarean delivery. They also had higher rates of hypertension, diabetes, renal disease, and foetal growth restriction. In addition, offspring born to women with HDP had significantly lower Apgar scores at 1 and 5 min. Increasing gravidity was also independently associated with lower odds of HDP. The inverse association between gravidity and HDP is consistent with previous evidence showing a higher risk of hypertensive disorders among women experiencing their first pregnancy (15).

UAE nationals and non-UAE nationals had comparable HDP rates. Despite similar prevalence, UAE nationals had a higher burden of co-morbidities than non-UAE nationals. This observation suggests that factors beyond genetic predisposition may be associated with HDP occurrence (16). Differences in HDP characteristics observed between the groups may reflect variations in demographic, lifestyle factors, or healthcare access disparities. Regional variation may also be influenced by small subgroup sample size, differences in insurance coverage among non-UAE nationals, or a referral patterns toward more severe cases at LWCH.

The WHO Eastern Mediterranean Region (EMR) women represented the largest proportion of our study sample and had the highest rates of hypertension (43.7%), diabetes (11%), renal disease (3.2%), and foetal growth restriction (0.8%). However, HDP prevalence was relatively low (4.4%). Additionally, the prevalence of PCOS among women from the EMR was 1.3%, which is comparable to another study from the region reporting a rate of 1.6% (17).

Certain patterns observed warrant further investigation. HDP prevalence was highest in WPR (9.1%), where women had the highest mean maternal age (37 years), followed by AMR (9.0%), where women had the highest mean BMI and the youngest mean maternal age (29.2 years). Although the AMR subgroup included only six HDP cases, resulting in potential unstable prevalence estimates, the observed pattern is broadly consistent with reports from WHO showing that the Region of the Americas had the highest prevalence (67.5%) of overweight and obesity globally in 2022 compared with all regions (18). However, given our small sample size, the findings should be considered exploratory. In contrast, SEAR had a lower prevalence of HDP and a lower mean maternal age (35 years) compared with several other regions.

A study conducted in Saudi Arabia showed that the prevalence of HDP is 2.4% and pre-eclampsia made up 54.9% of all HDP cases (19). The study reported that the prevalence of maternal complications was 9.4% and maternal mortality was 1.3%. They also reported that most cases were asymptomatic, which may contribute to challenges in HDP diagnosis. Other studies in the region reported a prevalence of pre-eclampsia varying from 0.17% to 5% (11).

HDP encompasses a spectrum of conditions which share several risk factors and clinical features, where patients may progress from one condition to another, making differentiation challenging (1). Therefore, we investigated their prevalence collectively, particularly given the overlap in diagnostic coding observed during our study.

Several co-morbidities commonly associated with HDP are more prevalent in the Middle East and North Africa (MENA) region. Diabetes mellitus affects 12.2% of adults in MENA compared with 8.3% globally (20). Nearly 40% of adults in EMR are hypertensive, compared with 32%-34% globally (19, 20).Consistent with these observations, hypertension was present in 43.7% of EMR women with HDP in our study, the highest among all regions. Mahmoud et al. further showed that metabolic syndrome is threefold more common among UAE nationals compared with global estimates (6).

HDP diagnosis is challenging due to variable clinical presentations (21). Klemmensen et al. estimated a prevalence of 3.27% in their registry through EMR coding, but upon aligning diagnoses with the gold standard guidelines from the American College of Obstetricians and Gynaecologists, the prevalence increased to 5.86% (22). Gestational hypertension was also significantly underestimated in their registry (0.55%) in comparison to the chart review (2.96%). The authors attributed this difference to misclassification, suggesting the potential limitations of relying on registry-based coding and possibly that HDP prevalence may be underestimated in similar settings.

Although the prevalence of HDP in our cohort (4.7%) lies within global estimates, it remains relatively high. Pre-eclampsia accounted for 2.3% in our study, exceeding the previously reported global prevalence of around 1% (23). A recent systematic and meta-analysis from Saudi-Arabia reported a pooled pre-eclampsia prevalence of 4.8%, supporting the observation that HDP continue to represent a substantial maternal health burden in the Gulf Region (24). In our study, we also observed an increase in pre-eclampsia prevalence from 1.8% in 2018 to 2.8% in 2023. This observation coincided with the COVID-19 pandemic, during which a meta-analysis showed that infected women with COVID-19 showed higher pre-eclampsia rates compared with uninfected women (7.0% vs. 4.8%) (25).

Beyond its immediate consequences, HDP is associated with an increased long-term burden of cardiovascular and metabolic disease among both mothers and offspring (8). Moreover, our results showed that no women with eclampsia had a documented prior diagnosis of HDP or pre-eclampsia. This finding may suggest late presentation to the hospital, under recognition of HDP, or challenges in screening and diagnosis. The associated public health and economic burdens are considerable. These findings suggest the importance of awareness campaigns, early screening programs, accessible prophylactic strategies, and close postpartum surveillance to support maternal and offspring health.

### 4.1 Limitations to the study

This study has several limitations that warrant consideration. First, although Latifa Women and Children Hospital (LWCH) is the main public maternity referral hospital in Dubai, a substantial proportion of deliveries in the emirate occur in private hospitals, often influenced by insurance coverage. This may introduce selection bias, as women delivering at LWCH may differ systematically from those attending private facilities, particularly in terms of socioeconomic background, healthcare utilisation, and severity of disease. In addition, as LWCH is a tertiary referral centre for severe and complicated obstetric cases, the study cohort may include a higher proportion of high-risk pregnancies than the wider UAE obstetric population. Accordingly, the prevalence estimate reported in this study should be interpreted as a hospital-based estimate rather than a nationally representative estimate for the UAE.

Second, HDP case identification relied on International Classification of Diseases (ICD) coding within the electronic medical record system. Although ICD coding provides a standardized approach for recording clinical diagnoses, coding inaccuracies, whether through misclassification or omission, may have led to underestimation or overestimation of HDP prevalence. In the absence of formal chart validation, some degree of misclassification cannot be excluded. Nevertheless, previous validation studies have shown acceptable performance of ICD-based HDP ascertainment, although accuracy may vary across settings (26, 27). For example, Labgold et al. demonstrated sensitivity >80% and positive predictive values >90% for ICD-10-defined HDP when compared with detailed chart review (26). Johnston et al. concluded in a systematic review that validated administrative definitions for HDP generally reported high specificity, although performance varied across settings (27). Future validation studies involving detailed chart review and reassessment of coding practices would help improve the accuracy of prevalence estimates and the classification of HDP subtypes.

Third, as with all retrospective studies based on routinely collected electronic medical records, incomplete or missing data cannot be excluded, which may introduce information bias and influence the precision of the analysis (28). Fourth, although the cohort was ethnically diverse, some subgroup analyses included relatively small numbers of HDP cases, which may have reduced the precision and reliability of subgroup-specific prevalence estimates. Finally, as this was a single-centre study, findings may not be fully generalizable to the broader UAE population due to differences in healthcare utilisation, referral patterns, and population demographics across healthcare settings.

## 5 Conclusion

Hypertensive disorders of pregnancy (HDP) remain an important maternal health concern in the United Arab Emirates. This study provides one of the first UAE-based estimates of HDP prevalence in a large multi-ethnic obstetric population. Although the prevalence fell within global ranges, HDP was associated with adverse maternal and neonatal outcomes. At a public health level, HDP has important implications for the long-term cardiovascular and metabolic health of mothers and their offspring. Strengthened screening, awareness campaigns, and accessible preventive measures are essential to reduce morbidity and improve maternal and neonatal outcomes.

## Data Availability

The data analyzed in this study is subject to the following licenses/restrictions: The datasets generated and analysed during the current study are available from the corresponding author on reasonable request. Requests to access these datasets should be directed to Aida.Azar@dubaihealth.ae.
